# Prognostic implication of 1-year decline in diffusing capacity in newly diagnosed idiopathic pulmonary fibrosis

**DOI:** 10.1038/s41598-024-59649-5

**Published:** 2024-04-17

**Authors:** Hyeonsu Lee, So Yeon Kim, Young Sik Park, Sun Mi Choi, Jong Hyuk Lee, Jimyung Park

**Affiliations:** 1 Division of Pulmonary and Critical Care Medicine, Department of Internal Medicine, Seoul National University Hospital, 101, Daehak-ro, Jongno-gu, Seoul, 03080 Republic of Korea; 2 Department of Radiology, Seoul National University Hospital, Seoul, Republic of Korea

**Keywords:** Idiopathic pulmonary fibrosis, Pulmonary diffusing capacity, Vital capacity, Disease progression, Respiratory function test, Respiratory tract diseases, Respiration

## Abstract

The progression of idiopathic pulmonary fibrosis (IPF) is assessed through serial monitoring of forced vital capacity (FVC). Currently, data regarding the clinical significance of longitudinal changes in diffusing capacity for carbon monoxide (DLCO) is lacking. We investigated the prognostic implications of a 1-year decline in DLCO in 319 patients newly diagnosed with IPF at a tertiary hospital between January 2010 and December 2020. Changes in FVC and DLCO over the first year after the initial diagnosis were reviewed; a decline in FVC ≥ 5% and DLCO ≥ 10% predicted were considered significant changes. During the first year after diagnosis, a significant decline in FVC and DLCO was observed in 101 (31.7%) and 64 (20.1%) patients, respectively. Multivariable analysis showed that a 1-year decline in FVC ≥ 5% predicted (aHR 2.74, 95% CI 1.88–4.00) and 1-year decline in DLCO ≥ 10% predicted (aHR 2.31, 95% CI 1.47–3.62) were independently associated with a higher risk of subsequent mortality. The prognostic impact of a decline in DLCO remained significant regardless of changes in FVC, presence of emphysema, or radiographic indications of pulmonary hypertension. Therefore, serial monitoring of DLCO should be recommended because it may offer additional prognostic information compared with monitoring of FVC alone.

## Introduction

Idiopathic pulmonary fibrosis (IPF) is a chronic fibrosing interstitial lung disease of unknown etiology, with a poor prognosis^1^. Although IPF is considered a progressive disease characterized by progressive worsening of lung function, its clinical course varies significantly among patients^2^. Therefore, it is necessary to regularly follow-up patients to assess disease severity. Assessment of IPF severity typically involves pulmonary function tests (PFTs) and chest imaging studies to evaluate the physiological and radiological aspects of the disease.

The forced vital capacity (FVC) remains the standard measure of pulmonary function in patients with IPF. Disease severity assessed using the baseline FVC correlates well with the future risk of mortality^3^. Therefore, in numerous clinical trials of IPF, the most commonly used primary endpoint is the longitudinal decline in FVC^4,5^. Particularly, a decline in FVC ≥ 10% predicted has been shown to be associated with a significantly increased risk of mortality in the subsequent period and has been widely used as a threshold for defining significant disease progression of IPF^6^. However, even a marginal decline in FVC, defined as a decline of 5–10%, has also been shown to be associated with poor outcomes^7^.

Although FVC possesses the advantage of being easily measured using spirometry, a more comprehensive evaluation beyond FVC measurement is required to assess the disease severity of IPF more accurately. In this context, diffusing capacity for carbon monoxide (DLCO) is another lung function parameter commonly used in IPF. The gender-age-physiology (GAP) index, the most universally used prognostic scoring system for IPF, includes both FVC and DLCO^8^. However, whether DLCO provides incremental information in addition to FVC for predicting the prognosis of patients with IPF remains unclear^3,9^. Additionally, data regarding the clinical significance of longitudinal changes in DLCO over time are limited^10^. Thus, this study aimed to investigate the prognostic implications of a decline in DLCO over the first year in patients newly diagnosed with IPF.

## Results

### Baseline patient characteristics

**Figure 1:**
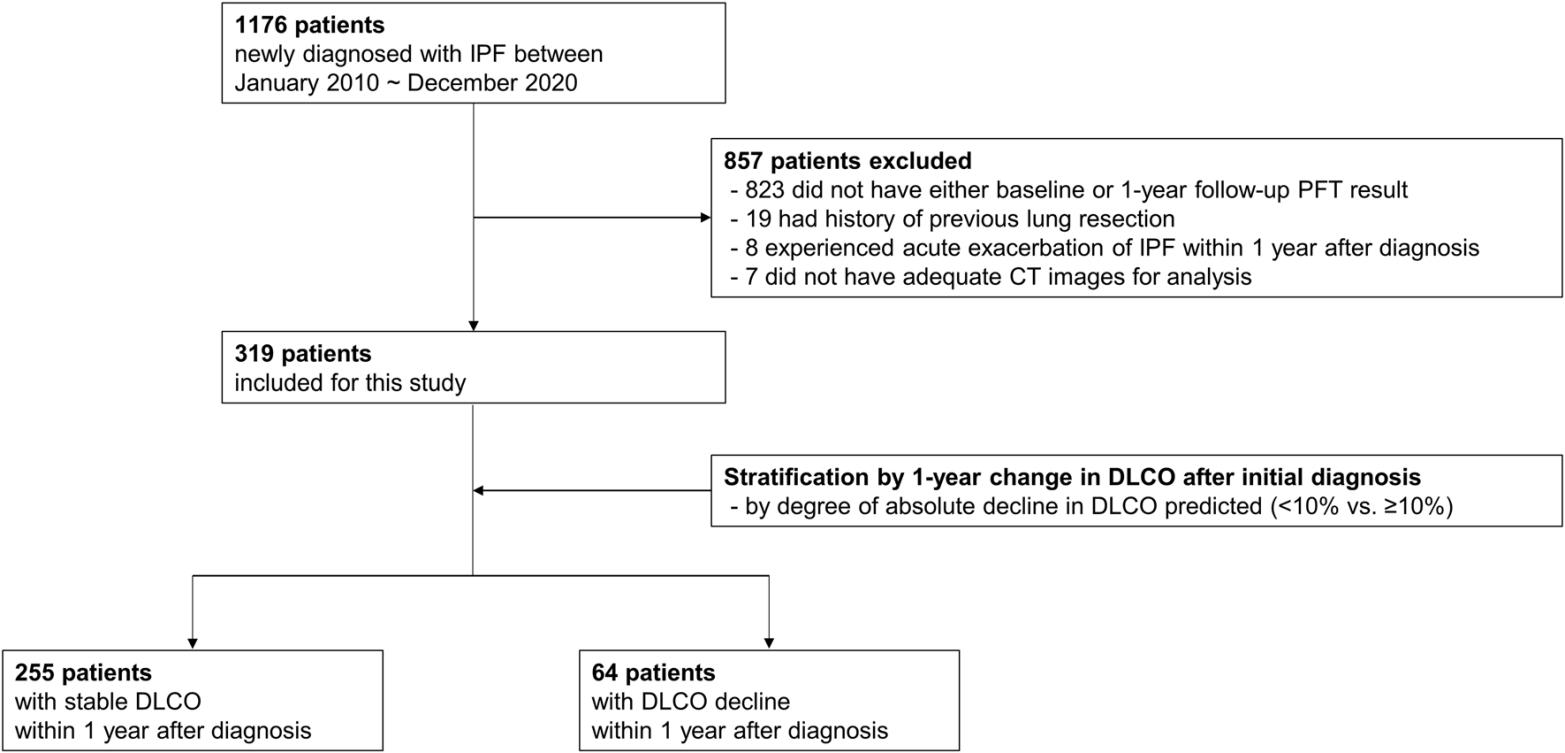
Study flow diagram showing stratification of patients by 1-year change in diffusing capacity for carbon monoxide (DLCO).

Between January 2010 and December 2020, 1176 patients were diagnosed with IPF. Among these patients, 857 were excluded; the most common reason for exclusion was the lack of PFT results (spirometry and DLCO measurements) at either baseline or 1-year follow-up. Consequently, 319 patients were included in this study and stratified into two groups based on the 1-year change in DLCO. Among the 319 patients, the median absolute decline in DLCO % predicted was 2% (− 4–8%). Specifically, 64 patients (20.1%) showed a decline in DLCO ≥ 10% predicted, while 255 patients (79.9%) indicated stable DLCO with a < 10% decline (Fig. 1).

The baseline demographics and PFT results are summarized in Table 1. The median patient age was 70 years. Most of the included patients were male with a history of cigarette smoking. The proportion of patients who started antifibrotic agents within 1 year of diagnosis did not differ between the two groups (60.0% vs. 57.8%, *P* = 0.750). Although the baseline FVC % predicted did not differ between the two groups (median 80.0% vs. 77.5%, *P* = 0.310), the baseline DLCO % predicted was higher in the group with a decline in DLCO than in the group with a stable DLCO (median 73.0% vs. 63.0%, *P* < 0.001).

**Table 1:** Patient characteristics according to change in DLCO over the first year.

	Stable DLCO (n = 255)	Decline in DLCO (n = 64)	*P*-value
Age, year	70 (65–75)	71 (66–77)	0.536
Sex, male	194 (76.1%)	55 (85.9%)	0.088
Body mass index, kg/m^2^	24.5 (22.6–26.8)	23.7 (22.0–25.7)	0.045
History of cigarette smoking	173 (67.8%)	44 (68.8%)	0.889
Comorbidities			
Hypertension	82 (32.2%)	17 (26.6%)	0.387
Diabetes	78 (30.6%)	11 (17.2%)	0.033
Cardiovascular disease	39 (15.3%)	5 (7.8%)	0.121
COPD	30 (11.8%)	1 (1.6%)	0.014
Use of antifibrotics within 1 year after diagnosis	153 (60.0%)	37 (57.8%)	0.750
Pulmonary function test			
FVC, L	2.7 (2.2–3.3)	2.7 (2.2–3.2)	0.954
FVC, % predicted	80.0 (69.0–92.0)	77.5 (66.0–91.0)	0.310
FEV_1_, L	2.2 (1.8–2.5)	2.2 (1.9–2.5)	0.125
FEV_1_, % predicted	91.0 (81.0–105.0)	94.0 (79.5–109.0)	0.442
DLCO, mL/min/mmHg	10.5 (8.4–13.0)	11.9 (9.1–14.5)	0.011
DLCO, % predicted	63.0 (50.0–74.0)	73.0 (56.0–85.0)	0.001
GAP score	3 (3–4)	3 (3–4)	0.174
Quantitative CT analysis			
Total lung volume, mL	3747.2 ± 966.4	3788.5 ± 852.9	0.755
Reticulation, %	7.6 (4.3–12.1)	9.8 (5.6–14.9)	0.029
Honeycomb, %	0.3 (0.0–2.2)	0.7 (0.0–3.5)	0.231
Ground glass opacity, %	1.0 (0.2–3.7)	0.8 (0.1–3.0)	0.334
Consolidation, %	0.2 (0.1–0.6)	0.3 (0.1–0.8)	0.105
Emphysema, %	0.2 (0.0–1.0)	0.2 (0.0–1.0)	0.916
Diameter of pulmonary artery and aorta on chest CT			
Pulmonary artery, mm	29 (26–31)	30 (27–32)	0.215
Aorta, mm	36 (33–39)	36 (34–39)	0.765
Pulmonary artery/aorta	0.79 (0.73–0.87)	0.81 (0.73–0.90)	0.235

Decline in DLCO is defined as an absolute decline in DLCO ≥ 10% predicted over the first year. Data are shown as mean ± SD, median (IQR), or number (%) as indicated.

*COPD* chronic obstructive pulmonary disease, *FVC* forced vital capacity, *FEV*_*1*_ forced expiratory volume in one second, *DLCO* diffusing capacity for carbon monoxide, *CT* computed tomography.

The results of the quantitative CT analysis are presented in Table 1. Among the several radiological abnormalities measured, only the extent of reticulation showed a between-group difference. The group with a decline in DLCO showed a greater extent of reticulation than in the group with a stable DLCO (median 9.8% vs. 7.6%, *P* = 0.029). The extent of emphysema was minimal in both groups, and there was no between-group difference (median 0.2% for both groups, *P* = 0.916).

The pulmonary artery-to-aorta ratio was measured using baseline chest CT images and showed a significant negative correlation with the baseline DLCO % predicted (r = − 0.282, *P* < 0.001). However, there was no significant difference in the pulmonary artery-to-aorta ratio between the group with a stable DLCO and the group with a decline in DLCO (median 0.79 vs. 0.81, *P* = 0.235). The proportion of patients with a pulmonary artery-to-aorta ratio > 0.9, which has been suggested as a threshold indicating the presence of pulmonary hypertension^11^, was 20.4% in the group with a stable DLCO and 26.6% in the group with a decline in DLCO (*P* = 0.284).

### Change in FVC and DLCO over 1 year after diagnosis

The interval between the initial diagnosis and 1-year follow-up PFT was 365 ± 35 days. The median absolute decline in FVC (mL and % predicted) over the first year was 50 mL (− 70–200) mL and 1% (− 3–6%), respectively. When a 5% decline was used as a cut-off, 101 of the 319 patients (31.7%) had an absolute decline in FVC ≥ 5% predicted.

**Figure 2:**
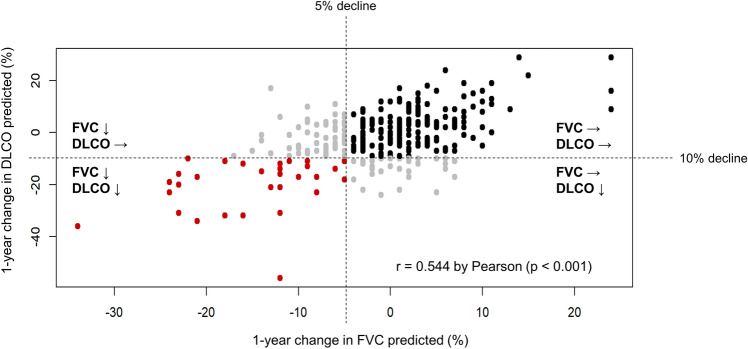
Significant correlation between change in forced vital capacity (FVC) and diffusing capacity for carbon monoxide (DLCO), but with exceptional cases of discrepancy between changes in FVC and DLCO.

A positive correlation was observed between the 1-year decline in FVC % predicted and DLCO % predicted (r = 0.544; Fig. 2). The proportion of patients showing a decline in FVC ≥ 5% predicted was higher in the group with a decline in DLCO than in the group with a stable DLCO (48.4% vs. 27.5%, *P* = 0.001). The mean 1-year decline in FVC % predicted was 6.8 ± 1.2% in the group with a decline in DLCO and 0.3 ± 0.4% in the group with a stable DLCO (*P* < 0.001).

However, FVC and DLCO did not always change in parallel in every patient. Some patients showed a discrepancy between changes in FVC and DLCO; 70 patients demonstrated a decline in FVC with a stable DLCO, and 33 patients demonstrated stable FVC with a decline in DLCO.

### Prognostic impact of a decline in DLCO

**Figure 3:**
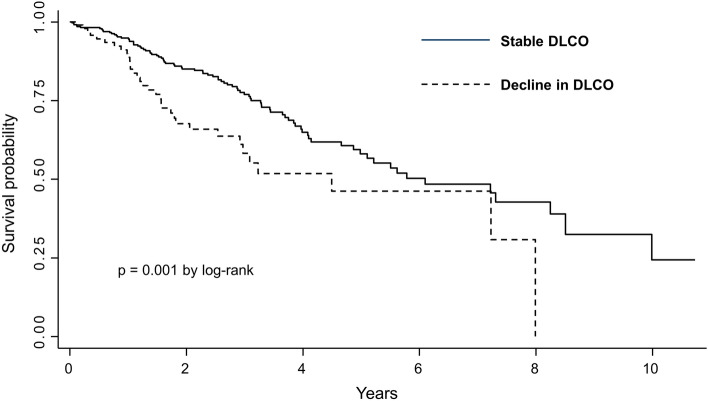
Kaplan–Meier estimates of time to death or lung transplant showing worse outcomes in patients with a decline in diffusing capacity for carbon monoxide (DLCO).

The median follow-up duration was 26 (13–42) months after the 1-year PFT examination. Of the 319 patients, 110 (34.4%) died, and 12 (3.7%) underwent lung transplantation. Time to death or lung transplantation was shorter in patients with a 1-year decline in DLCO ≥ 10% predicted than those with a stable DLCO. The median survival was 59 months for the group with a stable DLCO and 36 months for the group with a decline in DLCO (*P* = 0.001, log-rank test; Fig. 3).

By adjusting for pre-specified confounding variables, the multivariable analysis demonstrated that a 1-year decline in DLCO ≥ 10% predicted after the initial diagnosis was independently associated with a higher risk for future mortality (aHR 2.31, 95% CI 1.47–3.62). Similarly, the 1-year decline in FVC ≥ 5% predicted after the initial diagnosis was shown to be a significant risk factor for future mortality (aHR 2.74, 95% CI 1.88–4.00). Among other confounding variables, old age, male sex, lower baseline DLCO, and a greater extent of honeycombing on baseline chest CT images were associated with worse survival (Table 2).

**Table 2:** Association between 1-year decline in DLCO and risk for overall mortality.

	Univariable		Multivariable	
	Hazard ratio (95% CI)	*P*-value	Hazard ratio (95% CI)	*P*-value
Age, per 1 year	1.02 (1.00–1.04)	0.103	1.04 (1.02–1.06)	0.001
Male sex	1.51 (0.98–2.34)	0.063	1.95 (1.20–3.17)	0.007
Baseline lung function				
FVC % predicted, per 1%	0.98 (0.97–0.99)	0.003	0.99 (0.97–1.00)	0.067
DLCO % predicted, per 1%	0.98 (0.97–0.99)	< 0.001	0.97 (0.96–0.99)	< 0.001
Baseline CT characteristics				
Extent of reticulation, per 1%	1.04 (1.02–1.06)	0.001	1.00 (0.97–1.02)	0.829
Extent of honeycomb, per 1%	1.07 (1.03–1.11)	< 0.001	1.05 (1.01–1.09)	0.022
Change in lung function over the first year				
Decline in FVC ≥ 5%	2.33 (1.62–3.34)	< 0.001	2.74 (1.88–4.00)	< 0.001
Decline in DLCO ≥ 10%	1.92 (1.29–2.87)	0.001	2.31 (1.47–3.62)	< 0.001

Decline in FVC is defined as an absolute decline in FVC ≥ 5% predicted over the first year. Decline in DLCO is defined as an absolute decline in DLCO ≥ 10% predicted over the first year.

*CI* confidence interval, *FVC* forced vital capacity, *DLCO* diffusing capacity for carbon monoxide.

**Figure 4:**
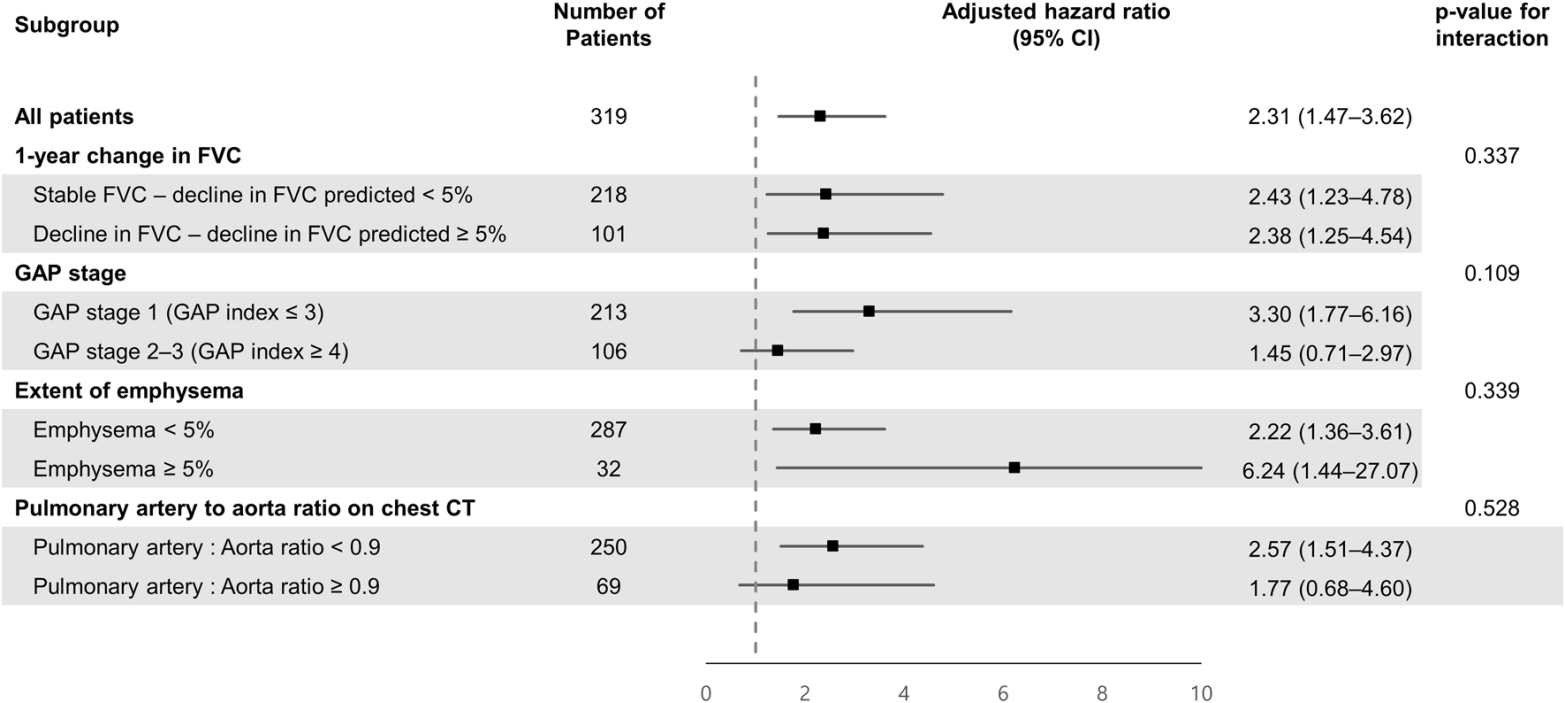
Subgroup analysis demonstrating the prognostic impact of a 1-year decline in diffusing capacity for carbon monoxide (DLCO).

In the subgroup analyses, the negative prognostic impact of the 1-year decline in DLCO was robust, regardless of the 1-year decline in FVC (Fig. 4). In 218 patients with a stable FVC (1-year decline in FVC < 5% predicted), aHR was 2.43 (95% CI 1.23–4.78), and in 101 patients with a decline in FVC (1-year decline in FVC ≥ 5% predicted), aHR was 2.38 (95% CI 1.25–4.54). The extent of emphysema or the pulmonary artery-to-aorta ratio determined using chest CT images did not influence the association between the 1-year decline in DLCO and worse survival.

## Discussion

In this study, we investigated the prognostic implications of a 1-year decline in DLCO after an initial diagnosis of IPF. Among patients newly diagnosed with IPF, approximately 20% showed a significant decline in DLCO, defined as an absolute decline in DLCO ≥ 10% predicted over the first year after the diagnosis. Although the extent of reticulation on chest CT images was greater in patients with a decline in DLCO than in those with a stable DLCO, most baseline characteristics were similar between the groups. This suggests that predicting whether a patient’s DLCO will decline or remain stable may be challenging at the time of initial diagnosis. The major finding of our study was that a significant decline in DLCO was associated with an increased risk of mortality, even after adjusting for other well-known prognostic factors of IPF, including age, sex, baseline FVC, and 1-year decline in FVC.

The clinical course of IPF varies significantly among patients^2^. Although IPF has traditionally been reported to have a median survival of 3 years from the time of diagnosis^12^, it is not always the case. In a previous meta-analysis that included only patients who had not been treated with antifibrotic therapy, marked variability was observed in the survival duration according to various study settings and designs^13^. This situation has become more complex with the advent of antifibrotic therapy. Although antifibrotic agents, including nintedanib and pirfenidone, have been shown to slow the progression of IPF in previous landmark randomized controlled trials^4,5^, the intraindividual response to antifibrotic therapy can be heterogeneous in the real world^14^. Therefore, it is imperative to closely monitor patients with IPF using comprehensive serial assessments.

In practice, FVC measurement using spirometry is the most popular method for monitoring patients with IPF because it is simple, inexpensive, and easily reproducible. However, the FVC alone cannot sufficiently predict the prognosis of patients with IPF. For example, a prior rate of decline in FVC has been depicted as a poor predictor of subsequent changes in FVC^15^. Thus, multidimensional evaluations beyond monitoring FVC alone are necessary to accurately assess IPF progression. Various assessment tools are used for this purpose, including the 6-min walk test, oxygen saturation test, health-related quality of life questionnaire, and quantitative analysis of chest CT images. In this study, we focused on DLCO because it is a relatively easy test to perform in most hospitals and has been standardized internationally^16^.

DLCO reflects the capacity of the lungs to exchange gas across the alveolar-capillary interface. It is the product of two parameters: (1) the rate constant for carbon monoxide uptake (KCO) and (2) the accessible alveolar volume (VA)^17^. As VA reflects the total lung capacity, unless there is an intrapulmonary airflow limitation, a natural correlation exists between DLCO and FVC, and a decline in DLCO is usually accompanied by a decline in FVC. However, as demonstrated by our results, some patients may show a decline in DLCO without a significant change in FVC. The association between a decline in DLCO and the risk of mortality was similar, even in patients with a stable FVC. We believe that this is because KCO, a component of DLCO, can be affected by several factors beyond lung volume, such as microvascular destruction, and temporal changes in these factors could be prognostically important independent of changes in FVC. Given the interindividual heterogeneity of physiological derangements in the lungs of patients with IPF, a decline in DLCO may capture different aspects of disease progression that cannot be detected by changes in FVC^18^. Therefore, serial measurement of not only FVC but also DLCO should be performed to monitor patients with IPF.

In light of our findings, careful consideration should be given to incorporating them into our clinical practice. In the scenario of patients with early stage IPF who are not receiving antifibrotic therapy, our study suggests that in addition to monitoring the trend in FVC, attention should also be paid to the trend in DLCO. While the trend in FVC remains a key factor in deciding whether to initiate antifibrotic agents^19^, our findings underscore the importance of assessing the trend in DLCO as well. Conversely, in patients actively treated with antifibrotic agents, the efficacy of these agents in slowing the decline in FVC has been well established^4,5^. However, if a patient exhibits a stable FVC with a substantial decline in DLCO, clinicians should be cautious and consider the possibility of poor clinical outcomes in the future. In such cases, therefore, closer monitoring may be required to mitigate adverse outcomes. Overall, our study highlights the importance of incorporating serial measurements of DLCO into the management of patients with IPF, both in treatment-naïve patients and in those receiving active antifibrotic therapy.

In patients with IPF, DLCO may be affected by the coexistence of emphysema or pulmonary hypertension. Patients with combined pulmonary fibrosis and emphysema (CPFE) present with significantly impaired DLCO and relatively preserved lung volume^20^. Thus, we evaluated whether the extent of emphysema affected the association between the change in DLCO and survival outcomes using subgroup analysis. We divided the patients using a cut-off value of 5% for the extent of emphysema, as suggested in a recent international statement^20^. Only a minority of patients (approximately 10%) had emphysema ≥ 5% in our study. Although statistical significance was not reached, which may be due to the small sample size, the aHR for death tended to be higher in patients with a greater extent of emphysema. This finding suggests that DLCO monitoring is important in patients with CPFE. However, we could not conduct a meaningful subgroup analysis based on the presence of COPD, defined by airflow obstruction on spirometry results, because the number of patients with COPD was too small (30 patients in the group with stable DLCO and only 1 patient in the group with a decline in DLCO). The underlying reason for this disproportionate distribution of concomitant COPD remains unclear. Regarding pulmonary hypertension, not every patient newly diagnosed with IPF underwent an echocardiographic evaluation at our center. Therefore, we measured the pulmonary artery-to-aorta ratio using chest CT images, which can be a reliable surrogate for pulmonary hypertension^11,21^. In our subgroup analysis, the prognostic impact of the decline in DLCO was robust regardless of the baseline pulmonary artery-to-aorta ratio.

Given the prognostic implications of a significant decline in DLCO, the next question in clinical practice is how to identify patients who will exhibit such a decline in DLCO in the future. In our study, compared with patients with a stable DLCO, those with a decline in DLCO showed a greater extent of reticulation on baseline chest CT images and had higher baseline DLCO % predicted. The observation of an inverse relationship between higher baseline DLCO and a more rapid decline over the first year is intriguing and difficult to interpret. However, a similar finding has also been noted in chronic obstructive pulmonary disease regarding the relationship between baseline forced expiratory volume in 1 s (FEV_1_) and the rate of decline in FEV_1_^22^. It is possible that some patients with lower baseline DLCO were unable to survive for up to 1 year or perform reliable DLCO measurements at follow-up. Another interesting finding is that the baseline extent of reticulation was greater in the group with a decline in DLCO. Although honeycombing is a hallmark of established fibrosis on chest CT images, the extent of reticulation may better reflect the ongoing fibrotic process. A few previous studies have also addressed the prognostic implications of the extent of reticulation not only in IPF but also in other types of interstitial lung diseases and even in interstitial lung abnormalities^23–25^. More studies employing quantitative analyses of chest CT images are required to determine whether this finding can be replicated.

The limitations of our study should be recognized to accurately appreciate the results. First of all, this was a retrospective study conducted at a single center. Given the limited sample size, we remain uncertain regarding the generalizability of our findings to other cohorts. Currently, we are actively involved in a nationwide effort to establish a multicenter cohort of patients with IPF in our country^26^. We believe that this collaborative effort will yield more robust and reliable data in the future. Second, we included only patients who had DLCO measurements at both baseline and the 1-year follow-up to calculate the rate of decline in DLCO, which may have introduced a selection bias. Patients with severe disease who could not undergo adequate DLCO measurements were excluded. Third, the change in DLCO during the first year after diagnosis could not accurately reflect the natural course of disease progression because it may have been influenced by the treatment received by the study patients. Finally, DLCO measurements are not entirely free from variability, even in a single laboratory setting^27^. However, considering that a 10% deviation has been used as a criterion for the repeatability of DLCO measurements^28^, we believe that an absolute decline in DLCO ≥ 10% predicted should be considered a clinically significant change.

In conclusion, a decline in DLCO ≥ 10% predicted over the first year after the initial diagnosis of IPF was associated with a higher risk of future mortality, and this prognostic impact was independent of a decline in FVC. Serial monitoring of DLCO may offer additional prognostic insights compared with monitoring of FVC alone, which could potentially influence early treatment decisions in patients newly diagnosed with IPF. Therefore, serial follow-up assessments of both FVC and DLCO are necessary for a more comprehensive evaluation of patients with IPF.

## Methods

### Study patients

This retrospective single-center cohort study examined patients newly diagnosed with IPF at Seoul National University Hospital, a referral tertiary hospital in South Korea, between January 2010 and December 2020. To investigate the prognostic impact of the initial 1-year decline in DLCO after the first diagnosis, only patients whose IPF diagnosis was initially established at our center were eligible for this study and screened. The diagnosis of IPF was established through multidisciplinary discussions according to the international guideline^29^. Patients referred to our center following their diagnosis at other centers were excluded. Among the eligible patients, those who were followed up for at least 1 year and underwent PFTs (spirometry and DLCO measurement) at baseline (within 3 months before and after diagnosis) and 1-year follow-up (between 9 and 15 months after diagnosis) were included in this study. Patients with a history of lung resection surgery or those who developed an acute exacerbation of IPF within the first year after diagnosis were excluded because these factors might complicate the interpretation of 1-year changes in PFT parameters. This study was approved by the Institutional Review Board of Seoul National University Hospital (IRB No. 2203-163-1310), and all methods were carried out in accordance with relevant guidelines and regulations. Requirement for informed consent was waived by the Institutional Review Board of Seoul National University Hospital because of retrospective observational study design.

### Study outcome and data collection

This study aimed to evaluate the prognostic implications of a 1-year decline in DLCO after the initial diagnosis of IPF. In particular, we investigated whether a decline in DLCO had an independent effect on the future risk of mortality after adjusting for a decline in FVC during the same period and other baseline confounders. Therefore, we compared the baseline and 1-year follow-up PFT results.

Given that most patients were at an early stage of the disease after the initial diagnosis, we applied the same threshold for defining a significant decline in lung function (FVC and DLCO), as suggested by the recent guideline for defining progressive pulmonary fibrosis^30^. Upon the 1-year follow-up evaluation, an absolute decline in FVC ≥ 5% and DLCO ≥ 10% predicted were considered significant changes in each parameter, and their prognostic implications were investigated. Thus, we divided patients based on whether they had an absolute decline in DLCO ≥ 10% predicted (group with a decline in DLCO) or not (group with a stable DLCO).

We retrospectively reviewed electronic medical records to gather information on patient demographic. PFT results were extracted from the PFT database at our center. Baseline chest computed tomography (CT) images were quantitatively analyzed using a lung texture analysis software (AVIEW Lung Texture version 1.1.43.7, Coreline Soft), and the extent of ground glass opacity, consolidation, reticulation, honeycombing, emphysema, and normal lung parenchyma were automatically calculated. As echocardiography is not routinely performed in every patient, we measured the pulmonary artery-to-aorta ratio as a surrogate marker for pulmonary hypertension using baseline chest CT images^11^. For mortality outcomes, we initially searched for the date of death by reviewing the medical records at our center. If this could not be identified, we used mortality data obtained from the Ministry of Public Administration and Security. Survival data were censored as of December 31, 2021. Patients who had undergone lung transplantation were considered as dead.

### Statistical analysis

Survival analysis was performed to investigate the impact of the 1-year decline in DLCO ≥ 10% predicted on mortality outcome. The index date was designated as the date of the 1-year follow-up PFT. The time to death or lung transplantation was compared between the two groups (stable DLCO vs. decline in DLCO) using the Kaplan–Meier method and the log-rank test. The Cox proportional hazards regression model was used for multivariable analysis to adjust for other confounding factors considered clinically significant, and adjusted hazard ratios (aHRs) with 95% confidence intervals (CIs) were calculated. Age, sex, baseline lung function (FVC and DLCO), baseline CT characteristics (extent of reticulation and honeycombing), and 1-year changes in FVC were selected as confounding factors.

Furthermore, we conducted a subgroup analysis according to the 1-year changes in FVC, baseline GAP stage, extent of emphysema, and pulmonary artery-to-aorta ratio to determine whether the prognostic impact of a decline in DLCO was robust. All statistical analyses were performed using the R statistical software version 4.2.0 (R Foundation for Statistical Computing, Vienna, Austria) and STATA software (version 17.0; StataCorp LP, College Station, TX, USA).

## Data Availability

The datasets used and/or analyzed during the current study are available from the corresponding author on reasonable request.

## Abbreviations

CI: Confidence interval
CPFE: Combined pulmonary fibrosis and emphysema
CT: Computed tomography
DLCO: Diffusing capacity for carbon monoxide
FEV_1_: Forced expiratory volume in 1 s
FVC: Forced vital capacity
GAP: Gender-age-physiology
HR: Hazard ratios
IPF: Idiopathic pulmonary fibrosis
KCO: Rate constant for carbon monoxide uptake
PFT: Pulmonary function test
VA: Alveolar volume
